# The effects of community environmental factors on obesity among Korean adults: a multilevel analysis

**DOI:** 10.4178/epih/e2014036

**Published:** 2014-12-24

**Authors:** Nan-He Yoon, Soonman Kwon

**Affiliations:** Graduate School of Public Health, Seoul National University, Seoul, Korea

**Keywords:** Obesity, Community environment, Multilevel analysis

## Abstract

**OBJECTIVES:**

This study explored multidimensional factors related to obesity by dividing them into individual and environmental factors, and performed multilevel analysis to investigate community environmental effects.

**METHODS:**

Data from the 2011 and 2012 Community Health Surveys were used for the analysis. Community-level variables, constructed from various regional statistics, were included in the model as environmental factors. Respondents with body mass index (BMI)≥25 were defined as obese, and a multilevel logistic regression analysis was conducted to analyze individual and environmental factors related to obesity. Moreover, a stratified analysis was conducted to compare factors related to obesity between men and women.

**RESULTS:**

Of 337,136 samples, 82,887 (24.6%) were obese, with BMI≥25. Sociodemographic characteristics at the individual level were mostly significantly related to obesity; however, while there were more obese men subjects among those with high socioeconomic status, there were more obese women among those with low socioeconomic status. There were fewer obese respondents among those who regularly walked and more obese respondents among those who reported short sleep duration or were highly stressed. At the community level, people living in areas with high socioeconomic status, high satisfaction with safety and public transportation, and high accessibility to sports facilities in their community had lower obesity risks.

**CONCLUSIONS:**

Community-level environmental factors affected obesity, especially perceived community environment, more significant than physical environment. Thus, it is necessary to develop effective obesity prevention and management strategies by considering potential community environmental factors that affect obesity.

## INTRODUCTION

As the obesity has been considered as a global public health issue, the obesity rates in South Korea have grown constantly for the last 10 years. According to the 2012 Korea National Health and Nutrition Examination Survey, the obesity rate among adults increased from 25.8% in 1998 to 32.8% in 2012 [1]. The obesity rate among children and adolescents also grew significantly, from 5.8% in 1998 to 15.3% in 2013 [2]. Moreover, according to a recent analysis of over 100 million health checkup cases in a big data study from the National Health Insurance Service, severe (BMI≥30) and morbid obesity rates (BMI≥35) increased 1.7- and 2.9-fold, respectively, in the last 10 years, higher rates of increase than the overall obesity rate [3]. These changes vary according to age and gender, which implies the need for targeted management plans.

Obesity increases the incidence and mortality of various chronic diseases such as hypertension, cardiovascular disorders, and diabetes. In particular, Asians tend to have higher fat ratios in their bodies even though they have lower weight or abdominal circumference than Caucasians, which increases their risks for chronic diseases due to obesity [4]. Accordingly, obesity increases individual medical uses and health expenditures [5], and the rising obesity rates also increase social costs. Total medical expenditures for 23 obesity-related diseases in South Korea increased to 12.7 trillion Korean won (KRW) in 2011, a 45.1% increase since 2007; similarly, national health insurance expenditure also increased by 40.5% to 2.9 trillion KRW in 2011 compared to 2007 [6].

Studies on obesity determinants and prevention and management strategies have mostly been conducted in North America where obesity was first described as a social problem. Recently, with increasing interest in obesity as a key public health issue in South Korea, more research has been conducted in various fields of study. However, most of these studies focus on individual factors, without an integrated perspective that includes interactions between individuals and their environments. Furthermore, many studies have been in small-scale, without sufficiently large sample sizes to be representative of populations [7].

Obesity is generally caused by an imbalance between caloric intake and consumption. However, the causes of obesity are still not clearly defined; it is determined by complex and multidimensional factors including genetics, health-related behavior such as diet and physical activity, and psychological and socioeconomic factors [8]. Previously, the causes of obesity were reported as individual genetic factors and health behaviors from a biomedical perspective; however, recent research on the complex causes of obesity has resulted in renewed discussions on the impacts of various environmental factors that are hypothesized to have a greater influence on obesity than genetic ones have [9-11].

The socioecological model offers a theoretical basis for the analysis of complicated and multidimensional factors that affect the obesity, as well as for the promotion of effective policies for its prevention and management [12]. Since individual behaviors are affected not only by individual beliefs but also by the surrounding environments, this model emphasizes the need to consider environmental factors and establish strategic guidelines to change those factors when implementing intervention strategies to change individual behavior [13].

As such, various articles have emphasized the need for a multidimensional approach in studies on health promotion and behaviors. Moreover, many researchers and policy makers are interested in developing comprehensive and multidimensional interventions and policies based on a socioecological perspective. In particular, strategies to solve various health problems discussed by international organizations including WHO since the 2000s as well as national health strategies deployed in the US such as Healthy People 2020 emphasize a socioecological approach that considers complex individual and environmental aspects.

Thus, this study aimed to explore multidimensional factors related to obesity by dividing them into individual and environmental factors based on the socioecological model, and to investigate the effects of community environment on obesity by applying multilevel analysis.

## MATERIALS AND METHODS

### Data

This study analyzed data from the 2011 and 2012 Community Health Surveys (CHS). CHS has been conducted annually in 253 health centers nationwide since 2008 with the purpose of establishing a foothold for carrying out evidence-based health programs by producing local health statistics and creating integrated evaluation indicators for health programs to be compared among regions. An average of five sample households are selected from each sample location in the survey, which is conducted through one-on-one interviews with all adult members of the household. CHS consists of both individual and household surveys. The individual survey asks questions about general sociodemographic characteristics, medical use, health status, and health behaviors. The household survey consists of questions about household income and residential type. CHS provides individual data from 253 communities nationwide, and enables researchers to consider various community-based administrative indicators as variables. This study developed variables using regional statistical data from CHS and public administrative statistics to distinguish and examine the influence of individual and environmental factors related to obesity.

The dependent variable in this study was obesity status, defined as BMI values of 25 or higher. BMI of each respondent was calculated as the respondent weight (kg)/[height (m)]^2^. The independent variables consisted of individual-level variables that might affect obesity including sociodemographic characteristics and health behaviors as well as socioeconomic, physical, and social environmental factors as community-level variables. As CHS is cross-sectional survey, the individual-level variables were collected from different respondents every year, however, the community-level variables were collected from the same 253 communities. Therefore, this study used community-level variables from the previous year to verify their influence on obesity. Among community-level variables, variables about satisfaction with social environment and social capital from CHS data were only surveyed in 2011. Thus, these variables from 2011 were included in the analysis.

Respondents who did not provide height and weight and outliers who reported height over 210 cm or weight over 180 kg were excluded from the analysis. Participants more than 65 years of age were also excluded from the analysis since the weight changes might be affected with other factors among the elderly. A total sample of 337,136 subjects were included in the analysis. Detailed descriptions and sources of variables are shown in Appendices 1 and 2.

### Data analysis

This study first examined the differences in distribution of obese participants according to sociodemographic characteristics and health behaviors using chi-square tests. Then, multilevel logistic regression analyses were conducted to investigate the effects of individual and environmental factors on obesity.

The formula for analysis is as follows:

logitPrYij=1Xij, Zj=γ00+γ01zj+γ10XijFixed part+Uoj+U1jXij+εijRandom partPr=probabilityYij=obesity status (BMI≥25 vs. <25)Xij=characteristics of i individuals living in j communityZj=characteristics of j communityγ01=community-level regression coefficientsγ10=individual-level regression coefficientsγ00=interceptU0j, U1j, εij=random error components

The empirical model consists of three phases. Model 1 was a null model with no explanatory variables to capture variations in interception between communities. Model 2, the effects of individual factors on obesity were analyzed by adding only individual-level variables and excluding fixed effects at the community level. For the third model, the impacts of environmental factors on obesity were analyzed by adding community-level variables.

Factors that affect obesity varied greatly by gender. Thus, the samples were stratified by gender and separately analyzed in the same model to compare the factors influencing obesity between men and women. All statistical analyses were conducted using SAS version 9.3 (SAS Institute Inc., Cary, NC, USA).

## RESULTS

### General characteristics of the sample

The general characteristics of the subjects are shown in Table 1. Of 337,136 respondents, 82,887 (24.6%) were obese, with BMI scores of 25 or higher. Significant differences were found in the distribution of obesity rates according to general characteristics (p<0.0001).

While 31.4% and 18.5% of man and woman respondents, respectively, were obese, the rates for both increased with age. Obesity rates were higher for lower income and education levels. Manual workers had an obesity rate of 27.4%, which was higher than non-manual workers. Respondents currently living with their spouses (26.4%) had higher obesity rates than those that did not (20.0%).

The distribution of obesity rates according to health behaviors revealed that former smokers had the highest rate (34.9%), followed by current smoker (29.3%) and those who never smoked (20.5%). Moreover, 25.7% of those who reported drinking alcohol at least once a month were obese, compared to 23.0% among those who did not. Subjects with moderate or vigorous physical activity as recommended had a higher obesity rate (26.1%) than those who did not (24.1%), whereas those who walked as recommended tended to have lower obesity rates (23.8%) than those who did not (25.2%). The obesity rate of participants on a low-sodium diet was 23.8%, lower than those who was not (25.1%). The subjects with longer sleep duration tended to indicate lower obesity rates. Moreover, 26.5% of subjects that responded that they were severely stressed were obese, a higher rate than those who were not (23.8%).

### Factors affecting obesity

Table 2 shows the results of multilevel logistic regression analysis that examined factors affecting obesity by dividing them into individual- and community-level factors. Model 1 examined community variations of obesity when the impacts of other independent variables were not considered, revealing significant differences in obesity rates among communities (p<0.0001). Variance at the community level was 0.022 (standard error [SE]=0.002), and the intraclass correlation coefficient (ICC) was 0.007, indicating that the community-level dispersion accounts for 0.7% of all dispersion (data not shown).

Model 2 examined the relationship between individual-level variables and obesity. There were more obese man respondents than woman, and the obesity rates increased with age and lower income and education levels. Non-manual workers had higher obesity rates than manual workers, and respondents currently living with their spouses had higher obesity rates than those who did not. Among health behaviors, current smokers showed lower obesity rates than those who never smoked, but higher obesity rates than former smokers. Those who reported drinking alcohol at least once a month showed a lower obesity rate than those who did not drink. There were no significant relationships between obesity and physical activity or low-sodium diet; however, respondents who reported walking as recommended had lower obesity rates than those who did not. Obesity rates were higher among respondents sleeping less than 7 hours compared to those sleeping an average of 7-8 hours a day, and those who reported severe stress were more obese that those who did not.

Model 3 examined the effects of community-level environmental factors on obesity after controlling for these individual factors. The model found that several environmental factors significantly influenced obesity. Among variables that represent community-level socioeconomic characteristics, people living in communities with high proportions of residents with at least a college degree tended to have lower obesity rates. Moreover, objectively measured physical environmental variables did not significantly influence obesity, but subjective perception of the community environment significantly influenced obesity. While obesity rates were higher among residents living in communities with high satisfaction with the natural environment, rates were lower among those living in communities reporting high satisfaction with use of public transportation.

### Factors affecting obesity according to gender

Tables 3 and 4 show the results of stratified multilevel logistic regression analysis by gender. Model 1 showed there were community variations in obesity, with significant differences in obesity rates among communities in both men and women (p< 0.0001) when the impacts of other independent variables were not considered. However, the community-level variance was greater for women (0.073, SE=0.007) with higher ICC (0.022) than men (0.014, SE=0.002) with lower ICC (0.004), also indicating that variation between communities was greater for women than men (data not shown).

Model 2 examined the relationship between individual-level variables and obesity, revealing gender differences in the effects of socioeconomic characteristics on obesity. The obesity rate increased with age for women, but did not have a significant influence on men after age 50. While men with lower income levels and education had lower obesity rates, women with these same traits had higher obesity rates. Man non-manual workers had higher obesity rates than those are not, whereas women showed higher obesity rates when they were manual workers or not in economic activity such as housewives or students. Both men and women had higher obesity rates when they live with their spouses.

Among health behaviors, both men and women former smokers had higher obesity rates than those who never smoked. Men who drank alcohol at least once a month were more obese than those who did not, whereas women who did not drink tended to be more obese. Differences in obesity were not significant for physical activities, but both men and women who walked as recommended had lower obesity rates. Low-sodium diets had differential impacts on obesity according to gender. While men on low-sodium diets were less obese, women tended to be more obese. Both men and women respondents sleeping less than 7 hours had higher obesity rates compared to those sleeping an average of 7-8 hours a day, and obesity rates were high for respondents of both genders who reported feeling severely stressed.

Model 3 examined the impacts of community-level environmental factors on obesity and also revealed gender differences. Among variables that represented community-level socioeconomic characteristics, women living in communities with high population densities with a college degree or higher tended to have lower obesity rates, while men did not. Moreover, physical environmental factors measured by objective variables did not have a significant influence on obesity among men, but women showed a low obesity rate if there were larger park areas within the community.

Among perceptions of social environment factors, both men and women living in communities with high satisfaction with the use of public transportation were less obese. Women living in communities with high accessibility to sports facilities were less obese, whereas men were not significantly influenced by this factor. Meanwhile, men living in communities with high satisfaction of safety tended to have lower obesity rates, while women were not significantly influenced by this factor.

## DISCUSSION

This study evaluated factors affecting the obesity of individuals from a socioecological perspective by dividing the factors into individual and regional levels. It found that individual-level sociodemographic characteristics were significantly related to obesity, and varied according to gender. Men generally showed a higher obesity rate with high socioeconomic status, whereas women with low socioeconomic status tended to be more obese. The relationship between health behaviors and obesity did not differ significantly between genders, but men on a low-sodium diet were less obese while women on a low-sodium diet were more obese. Both men and women who responded that they did not walk as recommended, slept for lesser durations, and were severely stressed, had higher obesity rates.

These results are generally consistent with previous findings that lower socioeconomic status increases obesity. This is due to the intake of high-calorie and low-nutrition foods or environments that limit time and access to regular physical activities [14,15]. However, this relationship varies according to social environments and context. While lower socioeconomic status in developed countries is linked to higher risks of obesity, higher socioeconomic status in developing countries is associated with higher risks of obesity [16,17]. This is because with rapid growth in economy and technology in developing countries, food choices and daily consumption symbolize material wealth [18]. This study also showed that the socioeconomic status resulted in different way for obesity between men and women. As men’s social activities are traditionally more active than women South Korea, men with high socioeconomic status are more likely to engage in high-intensity labor, leading to be less time for regular physical activity or selecting healthy foods. However, women with high socioeconomic status might easily use the facilities for weight controls in their communities. It is necessary to explore lifestyles and cognitive factors in more detail to verify these findings.

The risk of obesity was low among those living in communities where residents have high socioeconomic status, are more satisfaction with safety and public transportation, and have high accessibility to sports facilities. These results are similar to previous studies reporting that environments with factors that encourage healthy lifestyles lead to low obesity rates. Frank et al. [19] analyzed the correlation between obesity and community environments related to walking, and reported that individuals living in communities with higher walkability had low obesity rates. Community characteristics, such as population density, density of residential areas, diversity of land use, convenience of public transportation, installation of bike paths, accessibility to relevant facilities, and walkability, were reported as factors that affect obesity [19,20].

Recently, there have been increased efforts in South Korea to examine the correlation between physical environmental factors and health in urban and regional planning and geography. A study by the Korea Research Institute for Human Settlements [21] examined the impacts of physical environmental factors on the health of urban dwellers in order to build a healthy city. They found that community environmental factors, such as green space, extension of bike paths, mixed land use, the number of fast food restaurants, and the number of beds in hospitals, significantly influenced community as well as individual obesity rates. Furthermore, a study by Lee et al. [22] on Seoul citizens reported that physical environments based on neighborhoods such as mixed land use, neighborhood parks, and accessibility to streams affected the pedestrian activities of residents.

However, studies examining the impacts of environmental factors on obesity typically focus on physical environment factors, mostly using the same indicators as previous studies of North America. Objective indicators of physical environment factors such as the number of sports facilities or parks may not sufficiently explain the relationship between environment and obesity or obesity-related health behaviors [23]. This suggests that when measuring the influence of environmental factors, objective indicators and the subjective perceptions about their environments may differ [24]. According to Gebel et al. [25], the walking rate is lower and the obesity rate is higher if individuals feel that the environment is not suitable for walking even when objective measures indicate otherwise.

Seoul is a city with high walkability due to a high density of residential areas and mixed land use, and public transportation is well developed as well [22]. However, physical activity is relatively low and a decreasing number of people report walking activity, while the obesity rate continues to grow. Thus, this study examined the relationship between obesity and perceived community environments measured by subjective variables. The results showed that subjective perceptions were more significantly correlated with obesity than objective indicators of physical environmental factors were. It is important to explore the contexts of perceptions and experiences, through which such community environmental factors may affect obesity prevention and management. Further studies using qualitative methods are called for.

CHS data analyzed in this study were derived from a cross-sectional survey and thus were limited in explaining their correlation with obesity. Moreover, since obesity was determined by BMI that were calculated by self-reported height and weight, the true obesity rates may have been underestimated. However, this study offers an in-depth analysis on the impacts of community environmental factors on obesity by using regional administrative data from various sources as well as subjective perceptions of individuals surveyed in the same communities.

To effectively prevent and manage obesity, it is necessary to develop strategies that consider the effects of diverse environmental factors of communities. Previous studies have shown that obesity prevention programs that consider both public policy and environmental factors were more successful [9,26,27]. The current obesity-related policies and programs in South Korea focus mostly on improving individual behaviors for prevention and management [28], but this individual-centered approach has been shown to be limited in its effectiveness [29]. This is because changing diet habits and physical activities, which is particularly important for obesity prevention and management, also requires changing environmental factors that affect these behaviors. It is also necessary to improve the social norms that emphasize individual efforts and responsibilities to prevent and manage obesity.

By exploring and highlighting community environments related to obesity based on a socioecological perspective, this study will provide a theoretical basis to emphasize social responsibility to deal with the obesity issues and support efforts to develop more effective intervention strategies for obesity prevention and management.

## Figures and Tables

**Table 1.** General characteristics of subjects

	BMI< 25	BMI≥25	Total	p-value
Gender				< 0.001
Man	108,192 (68.6)	49,637 (31.4)	157,829 (46.8)	
Woman	146,057 (81.5)	33,250 (18.5)	179,307 (53.2)	
Age (yr)				< 0.001
19-34	68,371 (81.1)	15,913 (18.9)	84,284 (25.0)	
35-49	96,321 (74.7)	32,542 (25.3)	128,863 (38.2)	
50-64	89,557 (72.2)	34,432 (27.8)	123,989 (36.8)	
Household income				< 0.001
1st quartile (lowest)	30,822 (73.9)	10,865 (26.1)	41,687 (13.2)	
2nd quartile	72,662 (74.4)	25,024 (25.6)	97,686 (30.9)	
3rd quartile	67,732 (75.8)	21,647 (24.2)	89,379 (28.3)	
4th quartile (highest)	66,623 (76.6)	20,335 (23.4)	86,958 (27.5)	
Education				< 0.001
Middle school	56,649 (70.4)	23,765 (29.6)	80,414 (23.9)	
High school	108,324 (76.8)	32,774 (23.2)	141,098 (41.9)	
College or higher	88,757 (77.2)	26,186 (22.8)	114,943 (34.2)	
Occupation				< 0.001
Non-manual	62,983 (76.2)	19,713 (23.8)	82,696 (24.6)	
Manual	114,562 (72.6)	43,340 (27.4)	157,902 (46.9)	
Others	76,394 (79.5)	19,731 (20.5)	96,125 (28.5)	
Marital status				< 0.001
Live without spouse	76,737 (80.0)	19,183 (20.0)	95,920 (28.5)	
Live with spouse	177,283 (73.6)	63,647 (26.4)	240,930 (71.5)	
Smoking				< 0.001
Current smoker	57,654 (70.7)	23,874 (29.3)	81,528 (24.2)	
Former smoker	30,172 (65.1)	16,148 (34.9)	46,320 (13.7)	
Never smoker	166,371 (79.5)	42,854 (20.5)	209,225 (62.1)	
Alcohol drinking				< 0.001
< Once a month	106,684 (77.0)	31,881 (23.0)	138,565 (41.1)	
≥ Once a month	147,489 (74.3)	50,987 (25.7)	198,476 (58.9)	
Physical activity				< 0.001
No	193,778 (75.9)	61,627 (24.1)	255,405 (76.1)	
Yes	59,441 (73.9)	20,975 (26.1)	80,416 (23.9)	
Walking				< 0.001
No	147,368 (74.8)	49,536 (25.2)	196,904 (58.5)	
Yes	106,393 (76.2)	33,225 (23.8)	139,618 (41.5)	
Low-sodium diet				< 0.001
No	155,249 (74.9)	51,946 (25.1)	207,195 (61.5)	
Yes	98,988 (76.2)	30,940 (23.8)	129,928 (38.5)	
Sleep duration (hr)				< 0.001
<7	106,499 (73.4)	38,561 (26.6)	145,060 (43.0)	
7-8	138,985 (76.9)	41,719 (23.1)	180,704 (53.6)	
≥8	8,699 (77.1)	2,581 (22.9)	11,280 (3.3)	
Stress				< 0.001
Less severe	185,367 (76.2)	58,028 (23.8)	243,395 (72.2)	
Severe	68,805 (73.5)	24,839 (26.5)	93,644 (27.8)	
Total	254,249 (75.4)	82,887 (24.6)	337,136 (100.0)	

Values are presented as number (%).

BMI, body mass index.

**Table 2.** Factors affecting obesity based on multilevel analyses

		Model 2			Model 3		
		Estimate	SE	Pr > |t|	Estimate	SE	Pr > |t|
Fixed effects							
Individual level							
Gender (Ref: man)	Woman	-0.727	0.013	<0.001	-0.714	0.013	< 0.001
Age (Ref: 19-34, yr)	35-49	0.139	0.015	<0.001	0.142	0.015	< 0.001
	50-64	0.188	0.013	<0.001	0.189	0.013	< 0.001
Household income (Ref: 4th quartile, highest)	1st quartile	0.080	0.016	<0.001	0.083	0.017	< 0.001
	2nd quartile	0.069	0.012	<0.001	0.062	0.012	< 0.001
	3rd quartile	0.033	0.012	0.005	0.030	0.012	0.016
Education (Ref: college or higher)	Middle school	0.374	0.015	<0.001	0.366	0.016	< 0.001
	High school	0.037	0.011	0.001	0.032	0.012	0.007
Occupation (Ref: non-manual)	Manual	-0.032	0.014	0.019	-0.032	0.014	0.026
	Others	-0.019	0.012	0.130	-0.021	0.013	0.100
Marital status (Ref: live with spouse)	Without spouse	-0.265	0.011	<0.001	-0.262	0.012	< 0.001
Smoking (Ref: never smoker)	Current smoker	-0.040	0.014	0.003	-0.031	0.014	0.031
	Former smoker	0.162	0.015	<0.001	0.173	0.016	< 0.001
Alcohol drinking	≥Once a month	-0.046	0.010	<0.001	-0.046	0.010	< 0.001
Physical activity		0.000	0.010	0.965	-0.002	0.011	0.881
Walking		-0.061	0.009	<0.001	-0.062	0.009	< 0.001
Low-sodium diet		-0.002	0.009	0.793	-0.003	0.010	0.741
Sleep duration (Ref: 7-8, hr)	<7	0.155	0.009	<0.001	0.159	0.009	< 0.001
	≥8	0.033	0.025	0.180	0.034	0.026	0.191
Stress (Ref: less severe)	Severe	0.143	0.009	<0.001	0.153	0.010	< 0.001
Community level							
Population density					0.004	0.002	0.052
Fiscal self-reliance ratio					0.068	0.076	0.371
High education rate					-0.405	0.152	0.008
Park area					-0.854	0.490	0.082
Exercise facilities					0.083	0.097	0.395
Pedestrian safety					-0.018	0.060	0.760
Perceived accessibility to exercise facilities					-0.152	0.090	0.091
Satisfaction with safety					-0.370	0.216	0.088
Satisfaction with natural environment					0.266	0.127	0.037
Satisfaction with living environment					0.057	0.160	0.723
Satisfaction with public transportation					-0.298	0.087	0.001
Social support					-0.029	0.062	0.643
Nutrition education participation					0.511	0.285	0.073
Exercise program participation					0.172	0.242	0.477
Random effects							
o^2^		0.013	0.002	<0.001	0.009	0.001	<0.001

SE, standard error; Pr, probability; Ref, reference.

**Table 3.** Factors affecting obesity among men based on multilevel analysis

		Model 2			Model 3		
		Estimate	SE	Pr > |t|	Estimate	SE	Pr > |t|
Fixed effects							
Individual level							
Age (Ref: 19-34, yr)	35-49	-0.198	0.020	< 0.001	-0.198	0.021	<0.001
	50-64	-0.010	0.017	0.574	-0.009	0.018	0.605
Household income (Ref: 4th quartile, highest)	1st quartile	-0.086	0.022	0.000	-0.080	0.024	0.001
	2nd quartile	-0.039	0.016	0.013	-0.045	0.016	0.007
	3rd quartile	-0.034	0.015	0.025	-0.036	0.016	0.022
Education (Ref: college or higher)	Middle school	-0.069	0.020	0.001	-0.085	0.021	<0.001
	High school	-0.083	0.014	< 0.001	-0.093	0.015	<0.001
Occupation (Ref: non-manual)	Manual	-0.269	0.023	< 0.001	-0.262	0.024	<0.001
	Others	-0.063	0.015	< 0.001	-0.064	0.016	<0.001
Marital status (Ref: live with spouse)	Without spouse	-0.283	0.016	< 0.001	-0.283	0.017	<0.001
Smoking (Ref: never smoker)	Current smoker	-0.030	0.015	0.046	-0.015	0.016	0.337
	Former smoker	0.240	0.016	< 0.001	0.255	0.017	<0.001
Alcohol drinking	≥Once a month	0.030	0.014	0.034	0.025	0.015	0.091
Physical activity		-0.002	0.013	0.877	0.000	0.014	0.972
Walking		-0.084	0.012	< 0.001	-0.081	0.012	<0.001
Low-sodium diet		-0.036	0.013	0.005	-0.037	0.013	0.007
Sleep duration (Ref. 7-8, hr)	<7	0.142	0.012	< 0.001	0.148	0.012	<0.001
	≥8	0.032	0.036	0.375	0.043	0.038	0.256
Stress (Ref: less severe)	Severe	0.077	0.013	< 0.001	0.087	0.013	<0.001
Community level							
Population density					0.004	0.002	0.069
Fiscal self-reliance ratio					-0.046	0.089	0.606
High education rate					0.025	0.176	0.888
Park area					-0.375	0.565	0.507
Exercise facilities					0.081	0.113	0.470
Pedestrian safety					-0.031	0.070	0.662
Perceived accessibility of exercise facilities					-0.067	0.112	0.549
Satisfaction with safety					-0.594	0.251	0.019
Satisfaction with natural environment					0.294	0.148	0.047
Satisfaction with living environment					0.110	0.188	0.561
Satisfaction with public transportation					-0.242	0.101	0.017
Social support					0.082	0.072	0.255
Nutrition education participation					0.743	0.364	0.041
Exercise program participation					0.036	0.305	0.906
Random effects							
o^2^		0.013	0.002	< 0.001	0.010	0.002	<0.001

SE, standard error; Pr, probability; Ref, reference.

**Table 4.** Factors affecting obesity among women based on multilevel analysis

		Model 2			Model 3		
		Estimate	SE	Pr > |t|	Estimate	SE	Pr > |t|
Fixed effects							
Individual level							
Age (Ref: 19-34, yr)	35-49	0.464	0.024	<0.001	0.475	0.025	< 0.001
	50-64	0.367	0.021	<0.001	0.368	0.022	< 0.001
Household income (Ref: 4th quartile, highest)	1st quartile	0.247	0.023	<0.001	0.230	0.024	< 0.001
	2nd quartile	0.219	0.019	<0.001	0.205	0.020	< 0.001
	3rd quartile	0.125	0.019	<0.001	0.118	0.020	< 0.001
Education (Ref: college or higher)	Middle school	0.814	0.024	<0.001	0.804	0.026	< 0.001
	High school	0.332	0.019	<0.001	0.328	0.020	< 0.001
Occupation (Ref: non-manual)	Manual	0.192	0.022	<0.001	0.193	0.023	< 0.001
	Others	0.128	0.023	<0.001	0.123	0.024	< 0.001
Marital status (Ref: live with spouse)	Without spouse	-0.262	0.017	<0.001	-0.251	0.018	< 0.001
Smoking (Ref: never smoker)	Current smoker	0.051	0.037	0.171	0.051	0.039	0.186
	Former smoker	0.263	0.045	<0.001	0.263	0.047	< 0.001
Alcohol drinking	≥Once a month	-0.092	0.014	<0.001	-0.087	0.014	< 0.001
Physical activity		-0.027	0.016	0.102	-0.038	0.017	0.026
Walking		-0.062	0.014	<0.001	-0.068	0.014	< 0.001
Low-sodium diet		0.037	0.013	0.005	0.036	0.014	0.011
Sleep duration (Ref: 7-8, hr)	<7	0.102	0.013	<0.001	0.104	0.014	< 0.001
	≥8	0.152	0.034	<0.001	0.145	0.036	< 0.001
Stress (Ref: less severe)	Severe	0.193	0.014	<0.001	0.203	0.015	< 0.001
Community level							
Population density					0.004	0.003	0.103
Fiscal self-reliance ratio					0.195	0.103	0.059
High education rate					-1.072	0.209	< 0.001
Park area					-1.501	0.668	0.025
Exercise facilities					0.091	0.131	0.487
Pedestrian safety					-0.011	0.080	0.892
Perceived accessibility of exercise facilities					-0.270	0.125	0.032
Satisfaction with safety					-0.089	0.288	0.758
Satisfaction with natural environment					0.275	0.170	0.107
Satisfaction with living environment					-0.075	0.215	0.727
Satisfaction with public transportation					-0.332	0.116	0.005
Social support					-0.144	0.083	0.084
Nutrition education participation					0.399	0.409	0.329
Exercise program participation					0.399	0.345	0.248
Random effects							
o^2^		0.023	0.003	<0.001	0.013	0.002	< 0.001

SE, standard error; Pr, probability; Ref, reference.

## Appendix 1. Description of individual variables

**Table:**

Variable	Description	Source
Socio-demographic characteristics		Community health survey
Gender	Man, woman	
Age (yr)	19-34, 35-49, 50-64	
Household income	Household income quartiles	
Education	Middle school or under, high school, college or higher	
Occupation	Non-manual, manual, others (including students, housewives, not working)	
Marital status	Live with spouse, live without spouse	
Health behaviors		Community health survey
Smoking	Current smokers, former smokers, never smokers	
Alcohol drinking	More than once a month, less than once a month	
Physical activity	Moderate physical activity at least 30 minutes 5 times /wk or vigorous physical activity at least 20 minutes 3 times /wk	
Walking	Regular walking for at least 30 minutes 5 times per week	
Low-sodium diet	No salt or soy sauce in meals	
Sleep duration	Less than 7 hours, 7-8 hours, more than 8 hours	
Stress	Severe, less severe	

## Appendix 2. Descriptions of environmental variables at the community level

**Table:**

Variable	Description	Source
Economic environment		
Population density	1,000 persons per km^2^	Statistics of residence registration population
Fiscal self-reliance ratio	Ratio of own-source revenue to total local revenue	Municipal year book of Korea
High education rate	Population rate of university-or-higher education level	Population census
Physical environment		
Park area (km^2^)	Park area per total local area	Statistics of urban plan
Exercise facilities	Exercise facilities per 100 persons	Municipal year book of Korea
Pedestrian safety	Illegally parked car share at school zone, pedestrian fatalities per thousand people in traffic accidents	Traffic culture index
Perceived accessibility of exercise facilities	The percentage of participants that perceive accessibility of exercise facilities as easy	
Social environment		Community health survey
Satisfaction with safety	The percentage of participants satisfied with general safety (e.g., natural disaster traffic accident, crime)	
Satisfaction with natural environment	The percentage of participants satisfied with the natural environment (e.g., air, water)	
Satisfaction with living environment	The percentage of participants satisfied with the living environment (e.g., electricity, water and sewage, removal of garbage, sports facilities)	
Satisfaction with public transportation	The percentage of participants satisfied with the public transportation system (e.g., bus, subway, taxi)	
Social support Nutrition education participation	The percentage of participants that trusts and helps their neighbors Nutrition education participation rate at local public health centers, hospitals, or schools	
Exercise program participation	Exercise program participation rate at local public health or service centers	
