# Supplementary material for: The effects of community environmental factors on obesity among Korean adults: a multilevel analysis
Source: Epidemiol Health. 2014 Dec 24;36:e2014036. doi: 10.4178/epih/e2014036 (PMC4322521; doi:10.4178/epih/e2014036)
Supplement: Supplementary file 1 [file epih-36-e2014036-supplementary.pdf]

The Community Environment and Obesity: a multi-level analysis

Nan-He Yoon[a]; Soonman Kwon[a*]

[a] Graduate School of Public Health, Seoul National University

* Corresponding Author

Name: Soonman Kwon (Graduate School of Public Health, Seoul National University)

Address: Graduate School of Public Health, Seoul National University, 1 Gwanak-ro, Gwanak-gu, Seoul, 151-742, Korea

E-mail: kwons@snu.ac.kr

Phone: +82-2-880-2721

Fax: +82-2-762-9105

Abstract

Objectives

본 연구에서는 개인의 비만 발생에 영향을 미치는 다차원의 요인들을 개인적 요인과 환경적 요인으로 나누어 탐색 해 보고, 다수준분석 방법론을 적용하여 지역사회 환경이 개인의 비만 발생에 미치는 영향을 확인 해 보고자 하였다.

Methods

본 연구는 지역사회건강조사 2011, 2012년 자료를 활용하였으며, 보건복지부, 행정안전부 등의 다양한 지역 통계자료를 활용하여 지역 수준의 변수를 구성하였다. 체질량지수(BMI)가 25 이상인 경우를 비만으로 정의하고, 개인의 비만 발생에 영향을 미치는 개인적 요인과 환경적 요인의 효과를 구분하여 분석하기 위하여 다수준 로지스틱 회귀분석을 실시하였다. 또한 연구대상자를 성별에 따라 층화분석하여 남녀 간 비만 발생에 영향을 미치는 요인들의 차이를 확인하였다.

Results

전체 연구대상자 337,136명 중 BMI 25 이상의 비만인 응답자는 24.6%인 82,887명이었다. 개인수준의 인구사회학적 특성들은 대부분 비만 발생에 유의한 영향을 미치는 것으로 확인되었으나 남성의 경우 사회경제적 수준이 높은 응답자들에게서 비만이 더 많이 발생하는 것으로 나타난 반면, 여성의 경우에는 사회경제적 수준이 낮을수록 비만이 더 많이 발생하는 것으로 확인되었다. 또한 걷기를 실천하는 응답자들의 경우 비만이 더 적게 발생하는 것으로 나타났으며, 수면시간이 짧고 스트레스를 많이 받는다고 응답한 대상자들은 비만이 더 많이 발생하는 것으로 나타났다. 한편 지역수준에서는 사회경제적 수준이 높은 지역에 거주하는 경우, 지역 내 안전수준과 대중교통에 대한 만족도가 높은 지역

에 거주하거나 운동시설의 접근성이 높은 지역에 거주하는 경우 비만 발생의 위험이 적은 것으로 나타났다.

Conclusions

지역 수준의 환경적 요인들은 개인의 비만 발생에 영향을 미치는 것으로 확인되었으며, 특히 객관적인 지표로 측정한 물리적 환경 요인들에 대한 변수보다 각 환경적 특성들에 대한 주관적 인식과 비만 발생과의 연관성이 유의하게 나타났다. 이와 같이 개인의 비만 발생에 영향을 미치는 잠재적인 지역사회의 환경적 요인에 대한 탐색을 통해 다양한 차원의 환경적 특성과 배경, 맥락을 반영한 보다 효과적인 비만 예방 및 관리 전략을 개발하는 것이 필요하다.

Keywords

Obesity; Community Environment; Multilevel Analysis

Introduction

경제성장에 따라 소득수준이 증가하고 가공식품이나 외식의 증가 등 식생활환경이 변화하고 신체 활동량이 감소하는 등 개인의 생활습관과 환경 요인이 변화하면서 비만은 지속적으로 증가하고 있고 세계적으로 중요한 건강문제로 대두되고 있다. 세계보건기구(WHO; World Health Organization)에서는 비만을 21세기 신종 전염병으로 선포하였고, 매년 약 280만 명이 비만과 관련된 질병으로 사망하는 등 비만 문제는 점차 그 심각성이 증가하고 있다고 지적하였다(2000). 또한 주로 북미지역의 건강문제로 제기되었던 비만은 더 이상 특정 선진국만의 문제가 아닌 세계적인 문제이며, 현재 65% 이상의 국가에서 비만은 저체중보다 훨씬 심각한 건강문제를 초래하고 있다고 밝혔다[1].

우리나라에서도 지난 10여 년 간 성인의 비만율이 지속적으로 증가하였고, 아동과 청소년의 비만율도 빠르게 증가하고 있다. 2010년 국민건강영양조사 결과에 따르면 19세 이상 전체 성인인구 중 체질량지수(BMI; Body Mass Index)가 25 이상인 인구의 비율은 1998년 약 25.8% 였던 데 비해 2012년에는 32.8%까지 증가하였으며[2], 아동과 청소년의 2013년 비만율은 15.3%로 1998년의 5.8% 에 비해 급격히 증가하였다[3]. 또한 최근 국민건강보험공단의 건강검진 빅데이터 1억 여 건을 분석한 결과에 따르면, 고도비만율(BMI 30 이상)과 초고도비만율(BMI 35 이상)이 지난 10년 간 각 1.7배, 2.9배 증가한 것으로 나타나 전체적인 비만율의 증가보다 급격한 증가율을 보이는 것으로 나타났다[4]. 한편 이러한 비만율의 변화는 연령과 성별 등에 따라 다른 양상을 보이면서 증가하고 있어 대상에 적합한 관리 방안의 필요성이 강조되고 있다.

비만은 고혈압과 심혈관질환, 당뇨병 등 다양한 만성질환의 발생과 관련된 사망률을 증가시키는 것으로 알려져 있다. 특히 아시아인의 경우 서구에 비해 체중이나 복부둘레가 낮아도 만성질환 발생 위험을 증가시키는 체내 지방의 비율이 더 높은 경향이 있어 비만

이 초래하는 건강문제의 위험이 더 크게 나타날 수 있다[5,6]. 이로 인해 비만은 의료이용과 의료비 지출을 증가시키고[7], 비만의 증가에 따른 국가의 사회경제적 비용도 증가하고 있다. 서구 선진국에서는 비만과 관련된 질병에 소요되는 직접 비용이 총 국민의료비 지출의 2~7%를 차지하는 것으로 보고되고 있으며[8], 우리나라 국민의 비만 관련 질병 23개에 대한 총 진료비 지출 규모는 2011년 기준 12조 638억 원으로 2007년에 비해 5년 간 45.1% 증가하였고, 건강보험 급여비 지출 규모도 2011년 기준 2조 1,105억 원으로 2007년에 비해 40.5% 증가하였다[9].

또한 비만에 대한 사회적 관심의 증가와 함께 마른 체형을 강조하는 인식의 급격한 변화로 건강을 위한 적정체중의 유지 보다는 미(美)를 추구하기 위한 체중조절을 시도하는 경우가 점차 증가하고 있다[10]. 특히 자신의 건강상태를 고려하지 않은 부적절하고 무리한 체중조절 방법을 시도하는 것은 체중증가와 감소의 반복적인 악순환과 그로 인한 빈혈, 기초대사량 감소, 월경불순, 골다공증, 식이장애 등의 신체적 문제[11]와 자신의 신체에 대한 부정적인 태도와 그로 인한 우울증 등의 심리적인 문제[12,13] 등 2차적인 문제를 유발할 수도 있다. 이와 같이 우리나라에서도 비만이 중요한 보건학적 문제로 부각됨에 따라 비만예방과 관리를 위한 정책과 사업에 대한 관심도 증가하고 있으며, 효과적인 비만예방 및 관리를 위한 정책 및 전략 개발이 매우 중요한 시점이다.

비만의 결정요인이나 예방 및 관리 전략 등에 관련된 연구는 주로 비만이 가장 먼저 사회적 문제로 대두된 북미지역을 중심으로 활발하게 진행되어 왔다. 최근 우리나라에서도 비만에 대한 보건학적 관심이 증가하면서 다양한 학문 분야에서 비만과 관련된 연구가 점차 증가하고 있으나 대부분이 개인적 요인에 초점을 맞추고 있으며, 개인과 환경 간의 상호작용을 포함한 통합적 관점에서 다양한 차원의 요인들을 함께 고려한 연구는 미흡한 실정이다. 또한 주로 소규모의 개별 연구로 수행된 연구가 많았고, 대표성 있는 대규모의 표본 자료를 이용한 연구는 많지 않았다[10,14].

한편 일반적으로 비만은 에너지 칼로리 섭취와 소비 사이의 불균형으로 체지방이 과다하게 축적되어 발생한 것으로 설명된다. 그러나 비만의 원인은 여전히 명확하게 밝혀져 있지 않으며, 유전적 요인이나 식습관, 신체활동 등의 건강생활습관을 비롯하여 심리적인 요인, 사회 환경적인 요인 등 매우 복잡하고 다차원적인 요인들에 의해 결정되는 복합적인 문제로 알려져 있다[15,16]. 예전에는 비만의 원인을 주로 생의학적 관점으로 유전적인 요인과 건강행동 등 개인적 요인에서 찾았던 반면, 최근에는 비만의 복합적인 원인을 밝히고자 하는 연구가 활발하게 진행되면서 다양한 환경적 요인의 영향에 대한 논의가 계속되고 있으며, 일부 연구에서는 유전적 요인보다 환경적 요인의 영향력이 크다고 주장하기도 하였다[17-19].

사회생태학적 모형(Social ecological model)은 이와 같이 비만 발생에 영향을 미치는 복잡하고 다양한 차원의 요인들에 대한 분석과 효과적인 비만예방 및 관리 정책을 촉진하기 위한 이론적 기초가 될 수 있다[20]. 사회생태학적 모형은 개인의 행동은 개인의 신념 뿐 아니라 그를 둘러싼 다양한 환경요인에 의해 영향을 받으므로 환경적 요인과의 상호작용을 고려하는 것이 필요하며 개인의 행동을 변화시키기 위한 중재 전략을 수행할 때에도 환경적 요인을 함께 변화시킬 수 있는 전략 지침을 마련하는 것이 필요함을 강조하고 있다[21].

이와 같이 건강증진과 건강행동 연구에 대한 다차원적 접근의 필요성은 여러 문헌들을 통해 폭넓게 강조되고 있으며, 많은 연구자들과 정책결정자들이 사회생태학적 관점에 근거한 종합적이고 다차원적인 개입과 정책의 발전에 깊은 관심을 가지고 있다. 특히 2000년대 이후 WHO 등 국제기구에서 발표하는 다양한 건강문제 해결과 건강증진 달성을 위한 접근 전략과 미국의 Healthy People 2010 등과 같은 국가 차원의 보건정책 전략에서는 이와 같이 개인 수준과 환경 수준의 복합적인 측면을 고려하는 사회생태학적 접근을 강조하고 있다.

이에 본 연구에서는 사회생태학적 모형을 바탕으로 개인의 비만 발생에 영향을 미치는 다차원의 요인들을 개인적 요인과 환경적 요인으로 나누어 탐색해 보고, 다수준분석 방법론을 적용하여 지역사회 환경이 개인의 비만 발생에 미치는 영향을 확인해 보고자 한다.

Methods

연구대상 및 자료

본 연구는 지역사회건강조사 2011, 2012년 자료를 활용하여 분석을 실시하였다. 지역사회건강조사는 지역 건강통계를 생산하여 근거 중심의 보건사업 수행의 기반을 마련하고 지역 간 비교가 가능한 보건사업 통합평가지표를 생산하는 것을 목적으로 2008년부터 매 년 전국 253개 보건소에서 실시하고 있다. 조사는 1개 표본지점 당 평균 5개의 표본가구를 선정하여 만 19세 이상 성인 가구원 전수를 대상으로 1:1 면접조사 방식으로 실시된다. 지역사회건강조사는 개인조사와 가구조사로 구성되어 있으며, 개인조사는 흡연, 음주 등의 건강행태, 건강검진 및 예방접종, 이환, 의료이용, 사고 및 중독(손상), 활동제한 및 삶의 질, 보건기관 이용, 교육 및 경제활동 등으로 구성되어 있고, 가구조사는 가구소득, 거주형태 등으로 구성되어 있다. 이 자료는 전국 253개 시군구 단위의 개인 자료 활용이 가능하며 지역 단위의 다양한 행정지표들을 함께 변수로 고려할 수 있다는 장점이 있다. 따라서 본 연구에서는 지역사회건강조사 자료와 보건복지부, 행정안전부 등의 다양한 지역 통계자료를 활용하여 변수를 구성하고, 성인의 비만 발생과 연관된 개인적 요인과 환경적 요인을 구분하여 그 영향력을 살펴보았다.

본 연구의 종속변수는 비만 여부로, 각 응답자의 체질량지수(BMI = 체중(kg)/[신장(m)2])를 산출하여 체질량지수가 25 이상인 경우를 비만으로 정의하였다. 독립변수는 비

만 발생에 영향을 미칠 수 있는 잠재적인 개인요인들을 개인 수준의 변수(인구사회학적 특성, 건강수준 및 건강행동 등)로 포함하고, 잠재적인 환경요인들을 지역 수준의 변수(사회경제적 환경, 물리적 환경, 사회적 환경)로 포함하였다. 본 조사는 매 년 새로운 표본을 선정하여 조사를 실시하는 단면조사의 성격을 가지지만, 지역 수준으로는 매 년 동일한 253개 시군구를 대상으로 통계 자료 산출이 가능하다. 따라서 본 연구에서는 이전 년도의 지역 변수를 활용하여 개인의 비만 발생에 대한 영향을 확인하고자 하였다. 다만, 지역사회건강조사 자료를 통해 구성한 지역 수준의 변수 중 사회적 환경의 만족도에 대한 변수들(안전수준 만족도, 자연환경 만족도, 생활환경 만족도, 대중교통 만족도)과 사회적 자본의 측정 변수는 2011년도에만 조사가 이루어진 항목으로, 2011년도의 자료를 활용하여 변수를 구성하였다.

한편 체질량지수를 산출하기 위해 필요한 변수인 신장과 체중을 응답하지 않은 경우 혹은 극단치(신장 210cm 이상, 체중 180kg 이상)의 값을 응답한 경우는 분석 대상에서 제외하였다. 또한 65세 이상 노인의 경우에도 체중 변화의 양상과 영향요인이 다를 수 있으므로 분석 대상에서 제외하였다. 이렇게 선정된 최종 연구대상 337,136명을 대상으로 분석을 실시하였다.

구체적인 변수의 구성과 자료원은 표 1과 같다.

[Table 1 Here]

분석방법

본 연구에서는 먼저 연구대상자를 BMI 25 이상인 비만군과 BMI 25 미만인 정상군으로 구분한 후, 카이제곱 검정을 통해 각 집단에 포함되는 인구사회학적 특성과 건강수준 및 건강행동 실천 여부에 따른 연구대상자의 분포를 살펴보았다. 그리고 개인의 비만 발생에 영향을 미치는 개인적 요인과 환경적 요인의 효과를 구분하여 분석하기 위하여 다

수준 로지스틱 회귀분석을 실시하였다.

분석 식은 다음과 같다.

$$\text{logit}\{\Pr(Y_{ij} = 1|X_{ij}, Z_j)\} = \underbrace{\gamma_{00} + \gamma_{10}X_{ij} + \gamma_{01}Z_j + \gamma_{11}X_{ij}Z_j}_{\text{Fixed part}} + \underbrace{U_{1j}X_{ij} + U_{0j} + \varepsilon_{ij}}_{\text{Random part}}$$

단, Y_{ij}: 비만 여부 (BMI 25 이상/미만),

X_{ij}: *j* 지역에 거주하는 *i* 번째 개인의 특성, Z_j: *j* 지역의 특성

분석 모형은 총 3단계로 구성하였다. 첫 번째 모형에서는 지역 간 변이를 확인하기 위하여 독립변수를 모두 추가하지 않고 절편 만을 포함하는 기초모형을 분석하였다. 두 번째 모형에서는 지역 수준의 고정 효과를 제외한 개인 수준의 변수 만을 추가하여 개인적 요인이 개인의 비만 발생에 미치는 영향을 분석하였으며, 세 번째 모형에서는 두 번째 모형에 지역 수준의 변수를 추가하여 환경적 요인이 개인의 비만 발생에 미치는 영향을 분석하였다.

한편 비만의 발생 양상과 비만의 발생에 영향을 미치는 요인은 성별에 따라 큰 차이가 있으므로 연구 대상자를 성별에 따라 층화하고 동일한 모형의 분석을 실시하여 남녀 간 비만 발생에 영향을 미치는 요인들의 차이를 확인하였다. 모든 통계적 분석은 SAS 9.3 버전을 활용하였다.

Results

연구대상자의 일반적 특성

연구대상자의 일반적 특성은 표 2와 같다. 전체 연구대상자 337,136명 중 BMI 25 이

상의 비만인 응답자는 24.6%인 82,887명이었다. 일반적 특성에 따른 비만율의 분포는 모두 유의한 차이를 확인할 수 있었다(p<.0001).

전체 남성 응답자 중 31.4%가 비만으로 확인된 반면, 여성의 경우 18.5%로 적었으며, 연령이 높을수록 비만율이 높은 것으로 나타났다. 소득수준이 낮을수록, 최종학력이 낮을수록 비만율이 높았으며, 직업에 따라서는 비사무직인 경우 비만율이 27.4%로 사무직이나 기타 직업(주부, 학생 등)에 비해 높았다. 혼인상태에 따라서는 현재 배우자와 같이 거주하고 있는 응답자가 그렇지 않은 경우(20.0%)에 비해 비만율이 높은 것으로 확인되었다(26.4%).

한편 건강행동 실천 여부에 따른 비만율의 분포를 살펴보면, 흡연 여부에 따라서는 과거흡연자라고 응답한 대상자들 중 34.9%가 비만으로 나타나 비만율이 가장 높았고, 현재흡연자(29.3%), 비흡연자(20.5%)의 순으로 나타났으며, 월 1회 이상 음주를 하는 응답자의 경우 25.7%가 비만으로 나타나 그렇지 않은 경우(23.0%)에 비해 비만율이 높았다. 중등도 이상 신체활동을 권고안에 따라 실천하고 있는 대상자들의 경우 비만율이 26.1%로 신체활동을 실천하지 않는 대상자들(24.1%)보다 높았던 반면, 걷기를 권고안에 따라 실천하고 있는 대상자들의 비만율(23.8%)은 걷기를 실천하지 않는 대상자들의 비만율(25.2%)보다 낮아 다른 양상을 보였다. 저염식을 실천하고 있는 대상자들 중에는 23.8%가 비만으로, 저염식을 실천하고 있지 않은 대상자들의 비만율(25.1%)에 비해 낮았으며, 수면 시간에 따라서는 일 평균 수면시간이 길수록 비만율이 낮은 것으로 확인되었다. 또한 스트레스를 많이 받는 편이라고 응답한 대상자들 중에는 26.5%가 비만으로 확인되어 그렇지 않은 대상자들보다(23.8%) 높은 것으로 나타났다.

[Table 2 Here]

연구대상자의 비만 발생에 영향을 미치는 요인

다음은 연구대상자의 비만 발생에 영향을 미치는 요인을 개인 수준의 요인과 지역 수준의 요인으로 나누어 살펴 본 다수준 로지스틱 회귀분석의 결과이다(표 3). 먼저 다른 독립변수의 영향을 고려하지 않았을 때 비만 여부에 대한 지역별 변이를 확인해 본 연구모형 1에 따른 분석 결과, 시군구 별 비만율은 유의한 차이를 가지는 것으로 확인되었다 (p<.0001). 한편 지역 수준의 분산은 0.022(S.E=0.002)로, 전체 종속변수의 분산 중 지역 수준의 분산이 차지하는 비율을 확인하기 위해 급내상관계수(ICC: Intraclass Correlation Coefficient)를 산출 해 본 결과, ICC 값은 약 0.007로, 전체 분산 중 지역 수준의 분산이 차지하는 비율은 약 0.7% 정도 임을 알 수 있다.

개인 수준의 변수들과 개인의 비만 발생과의 관계를 살펴 본 연구모형 2의 분석 결과에 따르면, 여성에 비해 남성 응답자들에게 비만이 더 많이 발생하는 것으로 확인되었고, 연령이 높을수록, 소득수준과 최종학력은 낮을수록 비만이 더 많이 발생하는 것으로 나타났다. 직업에 따라서는 사무직에 비해 주부나 학생 등 기타 직업에 해당되는 경우 비만이 더 적게 발생하는 것으로 나타났고, 현재 배우자와 함께 거주하고 있는 경우 비만이 더 많이 발생하는 것으로 확인되었다. 건강행동 실천 여부에 따라서는 비흡연자에 비해 현재 흡연자는 비만 발생이 더 적은 것으로 나타났으나 과거흡연자에서는 더 많이 발생하는 것으로 나타났으며, 월 1회 이상 음주를 하는 경우 그렇지 않은 경우에 비해 비만이 적게 발생하는 것으로 확인되었다. 중등도 이상 신체활동이나 저염식 실천 여부에 따라서는 비만 발생의 차이가 유의하지 않았던 반면, 권고안에 따라 걷기를 실천하는 대상자들에서는 비만이 더 적게 발생하는 것으로 확인되었다. 또한 일 평균 수면시간이 7-8시간인 응답자들에 비해 7시간 미만 취침하는 응답자들에게서 비만이 더 많이 발생하는 것으로 나타났으며, 스트레스를 많이 받는다고 응답한 대상자들의 비만 발생이 더 많았다.

한편 이러한 개인적 요인들을 통제하였을 때 지역 수준의 환경적 요인들이 개인의 비만 발생에 미치는 영향을 살펴본 연구모형 3의 분석 결과, 모형에 포함된 일부 환경적 요인들이 개인의 비만 발생에 유의한 영향을 미치는 것으로 확인되었다. 지역 수준의 사회경제적 특성을 나타내는 변수들 중 대졸 이상 인구 비율이 높은 지역에 거주하는 대상자들일수록 비만이 적게 발생하는 것으로 나타났다. 또한 객관적 수준에서 측정한 물리적 환경의 변수들은 개인의 비만 발생에 유의한 영향을 미치지는 않는 것으로 나타났으나, 주관적 수준에서 측정한 사회적 환경의 변수들 중에는 지역 내 환경에 대한 주관적 인식 중 개인의 비만 발생에 유의한 영향을 미치는 변수가 확인되었다. 지역 내 자연환경에 대한 만족도가 높은 지역에 거주하는 주민들에게서는 비만이 더 많이 발생하는 것으로 나타난 반면, 지역 내 대중교통 이용에 대한 만족도가 높은 지역에 거주하는 주민들일수록 비만이 더 적게 발생하는 것으로 나타났다.

[Table 3 Here]

성별에 따른 비만 발생에 영향을 미치는 요인

다음은 다수준 로지스틱 회귀분석을 성별에 따라 층화하여 실시한 결과이다(표 4). 먼저 다른 독립변수의 영향을 고려하지 않았을 때 비만 여부에 대한 지역별 변이를 확인해 본 연구모형 1에 따른 분석 결과, 남성과 여성에서 모두 시군구 별 비만율은 유의한 차이를 가지는 것으로 확인되었다(p<.0001). 그러나 지역 수준의 분산은 남성의 경우 0.014(S.E=0.002), 여성의 경우 0.073(S.E=0.007)로 여성의 경우 지역 수준의 분산이 더 큰 것으로 확인되었으며, ICC 값도 남성에서는 0.004로 작았던 반면, 여성에서는 0.022로 크게 나타나 남성보다 여성에서 지역 간 변이가 더 큰 것을 알 수 있었다.

개인 수준의 변수들과 개인의 비만 발생과의 관계를 살펴 본 연구모형 2의 분석 결과

를 살펴보면, 사회경제적 특성들이 남성과 여성의 비만 발생에 영향을 미치는 방향에서 차이를 확인할 수 있었다. 여성의 경우 연령이 높을수록 비만이 증가하는 것으로 나타났으나 남성에서는 50세 이후로는 연령이 유의한 영향을 미치지 않는 것으로 나타났다. 남성의 경우 소득수준과 최종학력이 낮을수록 비만이 적게 발생한 반면, 여성의 경우에는 소득수준과 최종학력이 낮을수록 비만이 많이 발생하는 것으로 확인되었으며, 직업에 따라서도 남성은 사무직에 비해 비사무직이나 기타 직업인 경우 비만이 더 적게 발생하였으나 여성의 경우 사무직에 비해 비사무직이나 기타 직업인 경우 비만이 더 많이 발생하는 것으로 확인되었다. 혼인상태에 따라서는 남녀 모두에서 배우자와 함께 거주하고 있는 경우 비만이 더 많이 발생하는 것으로 확인되었다.

건강행동 실천 여부에 따라서는 남녀 모두에서 비흡연자에 비해 과거흡연자의 경우 비만이 더 많이 발생하는 것으로 나타났으며, 월 1회 이상 음주를 하는 남성의 경우에는 그렇지 않은 경우에 비해 비만이 더 많이 발생하는 반면, 여성의 경우에는 음주를 하지 않는 경우에 비만이 더 많이 발생하는 것으로 확인되었다. 권고안에 따른 중등도 이상 신체활동의 실천 여부에 따라서는 비만 발생의 차이가 유의하지 않았던 반면, 권고안에 따라 걷기를 실천하는 대상자들에서는 남녀 모두 비만이 더 적게 발생하는 것으로 확인되었다. 저염식 실천 여부는 성별에 따라 비만 발생에 다르게 영향을 미치는 것을 확인할 수 있었는데, 남성의 경우 저염식 실천을 하는 경우 비만이 더 적게 발생하였지만, 여성의 경우 저염식 실천을 하는 경우 비만이 더 많이 발생하는 것으로 나타났다. 한편 일 평균 수면시간이 7-8시간인 응답자들에 비해 7시간 미만 취침하는 응답자들에게서 남녀 모두 비만이 더 많이 발생하는 것으로 나타났으며, 스트레스를 많이 받는다고 응답한 대상자들의 비만 발생도 남녀 모두에서 더 많았다.

지역 수준의 환경적 요인들이 개인의 비만 발생에 미치는 영향을 살펴 본 연구모형 3의 분석 결과에서도 성별에 따른 차이를 확인할 수 있었다. 지역 수준의 사회경제적 특성

을 나타내는 변수들 중 대졸 이상 인구 비율이 높은 지역에 거주하는 여성들은 비만이 적게 발생하는 것으로 나타났으나 남성에서는 지역 수준의 사회경제적 특성에 따른 차이를 살펴볼 수 없었다. 또한 객관적 수준에서 측정한 물리적 환경 변수들의 경우 남성의 비만 발생에는 유의한 영향을 미치지 않는 것으로 나타났으나 지역 내 공원의 면적이 넓을수록 여성의 경우에는 비만이 더 적게 발생하는 것으로 확인되었다. 또한 주관적 수준에서 측정한 사회적 환경의 변수들 중에서는 지역 내 대중교통 이용에 대한 만족도가 높은 지역에 거주하는 주민들일수록 남녀 모두 비만이 적게 발생하는 것으로 나타났다.

한편 운동시설에 대한 주민들의 접근성이 높은 지역에 거주하는 여성들은 비만이 더 적게 발생하는 것으로 나타난 반면, 남성에게는 유의한 영향을 미치지 않는 것으로 나타났다. 지역 내 환경에 대한 주관적 인식 역시 성별에 따른 비만 발생에 다른 영향을 미치는 것으로 확인되었는데, 지역 내 안전수준에 대한 만족도가 높은 지역에 거주하는 남성들은 비만이 더 적게 발생하는 것으로 나타났으나 여성에게는 유의한 영향을 미치지 않았다.

[Table 4 Here]

Discussion

본 연구에서는 사회생태학적 관점에 따라 개인의 비만 발생에 영향을 미치는 요인들을 개인수준의 요인들과 지역수준의 환경요인들로 구분하여 살펴보았다. 2011년과 2012년 지역사회건강조사 자료와 다양한 자료원을 통해 구성한 개인과 지역수준의 변수들을 포함한 다수준분석 방법론을 적용하여 다차원적 비만 발생 영향요인들을 확인하였고, 성별에 따른 영향요인의 차이를 비교 분석하였다.

분석 결과, 개인수준의 인구사회학적 특성들은 대부분 비만 발생에 유의한 영향을 미치는 것으로 확인되었으나 이는 성별에 따라 매우 다른 양상을 보였다. 남성의 경우 대체로 사회경제적 수준이 높은 응답자들에게서 비만이 더 많이 발생하는 것으로 나타난 반면, 여성의 경우에는 사회경제적 수준이 낮을수록 비만이 더 많이 발생하는 것으로 확인되었다. 건강행동의 실천 여부와 비만 발생과의 관계는 일정한 경향을 보이지는 않았으나 권고안에 따라 걷기를 실천하는 응답자들의 경우 남녀 모두에서 비만이 더 적게 발생하는 것으로 나타났으며, 수면시간이 짧고 스트레스를 많이 받는다고 응답한 대상자들은 남녀 모두 비만이 더 많이 발생하는 것으로 나타났다. 저염식을 실천하고 있는 남성 응답자들은 비만 발생 위험이 더 적은 것으로 확인되었으나 여성들의 경우 저염식을 실천하고 있다고 응답한 대상자들에게서 비만이 더 많이 발생하는 것으로 확인되어 다른 양상을 보였다.

이는 대체로 선행연구의 결과와도 일치하는 결과로, 일반적으로 사회경제적 수준이 낮을수록 비만은 증가하는 결과가 확인되는데, 이는 고열량 저영양 식품을 주로 섭취한다거나 규칙적인 신체활동을 실천할 수 있는 시간이나 장소에 대한 접근성이 부족한 환경에서 기인한다고 볼 수 있다[22,23]. 그러나 이러한 관계는 사회의 환경이나 맥락에 따라 다른 양상을 보이는데, 주로 선진국에서는 사회경제적 수준이 낮을수록 비만 발생위험이 높은 것으로 확인된 반면, 개발도상국에서는 사회경제적 수준이 높을수록 비만 발생위험이 높은 것으로 알려져 있다[24,25]. 이는 개발도상국의 경우 급격한 경제와 기술의 발전과 함께 식품의 선택이나 일상 중 에너지의 소비가 물질적인 부를 상징하는 의미를 가지는 경우가 많기 때문으로 알려져 있다[26]. 본 연구의 분석 결과 남성과 여성의 사회경제적 수준에 따른 비만 발생 위험이 상이하게 나타난 것도 이러한 현상과 유사하게 남성의 사회활동이 활발한 한국 사회의 특성에 따라 사회경제적 수준이 높은 남성의 경우 노동강도가 높고 규칙적인 신체활동의 실천이나 건강한 식품의 선택이 어려운 환경에 노출되

는 경우가 많기 때문인 것으로 볼 수 있으나 생활습관이나 인지적 요인에 대한 추가적인 탐색이 필요하다.

한편 지역수준의 환경적 요인들의 경우 역시 성별에 따라 일정한 경향을 보이지는 않았다. 사회경제적 수준이 높은 지역에 거주하는 경우 비만 발생의 위험이 적은 것으로 확인되었고, 지역 내 안전수준과 대중교통의 접근성이나 이용편의성 등에 대한 만족도가 높은 지역에 거주하는 경우, 운동시설의 접근성이 높은 지역에 거주하는 경우 비만 발생의 위험이 적은 것으로 나타났다.

이러한 분석 결과는 지역 내 환경적 요인들이 건강한 생활습관을 실천하기에 유리한 환경일수록 비만율이 낮은 것으로 확인되는 선행연구들의 결과와도 유사한 경향을 보인다고 볼 수 있다. 걷기와 관련된 지역사회 환경과 비만 간의 관련성을 인구사회학적 특성의 차이에 따라 분석한 Frank 등(2008)의 연구 결과에 따르면, 주택지 밀집도가 크고, 거리 간 이동이 용이하며 용지 활용이 걷기를 실천하기에 유리한 지역일수록 비만 인구가 적은 것으로 확인되었으며, 인구밀도와 거주지역 밀집도, 토지 이용의 다양성, 대중교통 편의성, 자전거 도로 설치 여부, 관련 시설 접근성, 걷기와 관련된 지역사회의 도보 이동 용이성 등의 지역적 특성도 지역 주민의 비만과 비만관리 행동에 영향을 미치는 요인으로 보고되고 있다[27-30].

최근 국내에서도 도시·지역계획학이나 지리학 등의 학문 분야에서 도시 내 물리적인 환경요인들과 건강과의 연관성을 확인하고자 하는 시도가 점차 증가하고 있다. 국토연구원(2010)의 연구에서는 건강도시 구현을 위해 도시의 물리적 환경요인들이 도시민들의 건강상태에 미치는 영향을 살펴보았으며, 녹지공간의 면적과 자전거도로 연장, 토지이용 혼합도, 패스트푸드점 수, 병상 수 등의 지역의 환경요인들이 지역의 비만율과 개인의 비만 발생에 유의한 영향을 미치는 것으로 확인되었다[31]. 또한 서울시민들을 대상으로 실

시된 이경환 등(2007)의 연구에서는 토지이용 혼합도와 근린공원, 하천 접근성 등 근린 단위의 물리적 환경이 주민의 걷기 실천에 영향을 미친다고 밝히고 있다[32].

그러나 이와 같이 비만과 환경적 요인들의 영향을 살펴 본 연구에서 주목하고 있는 요인들은 주로 물리적 환경요인에 그치고 있었으며, 대부분 북미 지역을 중심으로 이루어진 선행연구들의 지표를 활용하여 그 영향력을 측정하고 있다. 그러나 최근 이러한 물리적 환경요인들을 측정하는 객관적인 지표들(예: 체육시설 수, 공원면적 등) 만으로는 환경과 비만, 혹은 비만 관련 건강행태와의 관계를 충분히 설명하지 못한다는 주장이 제기되고 있다[33]. 이는 환경요인의 영향력을 측정할 때 객관적인 지표들과 실제로 개인이 환경에 대해서 가지는 주관적 인식이 미치는 영향이 각기 다를 수 있다는 것을 의미한다[34]. Gebel 등(2011)의 연구에 따르면, 주변 근린환경이 객관적인 지표로 측정했을 때는 걷기에 유리한 환경이라고 하더라도 개인이 그 환경을 걷기에 부적절하다고 느끼는 경우 실제로 걷기 실천률이 더 낮고 비만인 경우도 더 많은 것으로 나타났다[35].

국내에서도 서울시의 경우 대중교통이 발달해 있고 주거 밀도와 토지이용 혼합도가 높아 일반적으로 보행친화성(walkability)이 높은 지역의 특성을 지니고 있으나[32] 신체활동 실천율이 상대적으로 낮고, 걷기 실천율은 지속적으로 감소하며 비만율은 지속적으로 증가하고 있는 것으로 보고되고 있다. 이에 본 연구에서는 지역 내 환경에 대한 주민들의 주관적 인식을 측정한 변수들을 지역수준의 환경적 요인 변수로 구성하여 비만 발생과의 연관성을 확인하였으며, 분석 결과 객관적인 지표로 측정한 물리적 환경 요인들에 대한 변수보다 각 환경적 특성들에 대한 주관적 인식과 비만 발생과의 연관성이 더 큰 것으로 나타났다. 향후 실제로 이러한 지역의 환경요인들이 어떠한 인식과 경험 등의 맥락을 통해 개인의 비만예방·관리 행동 실천에 영향을 미칠 수 있는지 그 과정에 대한 탐색이 중요하며, 질적 연구 방법론 등을 적용한 추가 연구가 필요할 것이다.

본 연구에서 분석한 지역사회건강조사 자료는 단면조사의 특성을 가지므로 건강행동의 실천 여부 등과 같은 개인수준의 변수들에 대해서는 비만 발생과의 선후 관계를 확인할 수 없으므로 비만 발생과의 연관성을 설명하기에 한계가 있으며, 자가 보고한 신장과 체중으로 산출한 체질량지수를 기준으로 비만 여부를 판단하였기에 비만율이 과소 추정될 가능성이 있다는 한계점을 가진다. 그러나 본 연구에서는 동일한 지역을 기준으로 패널 형식으로 구축된 자료를 통해 개인들을 대상으로 조사된 주관적 인식을 비롯하여 다양한 자료원의 지역 행정자료를 함께 활용하여 지역사회 환경 요인들이 개인의 비만 발생에 미치는 영향에 대하여 보다 심층적인 분석이 가능하다는 점에서 의미가 있다고 볼 수 있다.

효과적인 비만 예방과 관리를 위해서는 이와 같이 다양한 환경적 요인의 영향을 고려한 전략의 구상이 필요하다. 실제 연구결과에서도 정책 및 환경적 요인의 중재 전략을 함께 고려한 비만 예방 프로그램들이 보다 성공적이었던 것으로 확인되었다[36-38]. 우리나라의 기존 비만 관련 정책 및 사업들은 주로 개인의 행동 개선을 통한 예방 및 관리에 초점이 맞추어져 있으나[39] 비만예방 및 관리를 위한 개인 중심의 접근 방법은 효과가 그리 높지 않은 것으로 알려져 있다[40]. 비만 예방 및 관리에 특히 중요한 식습관과 신체활동의 개선은 그에 영향을 미치는 환경적 요인들을 변화시키는 노력을 반드시 포함하여야 효과가 있을 수 있기 때문이다. 이와 같이 개인의 비만 발생에 영향을 미치는 다양한 환경적 요인들에 대한 고려와 그를 포함한 중재 전략의 개발에 대한 필요성은 국내외 많은 선행연구들을 통해 강조되고 있다. 그러나 국내의 비만 관련 정책은 주로 개인의 행동 개선에만 초점이 맞추어져 있어 많은 한계가 지적되고 있으며, 비만의 예방 및 관리는 모두 개인의 책임으로 인식되는 사회적 분위기에 대한 개선도 필요하다.

이에 본 연구와 같이 사회생태학적 관점을 바탕으로 비만 발생과 연관된 지역사회의 환경에 대한 탐색을 통해 다양한 차원의 환경적 특성과 배경, 맥락을 반영한 중재 전략을

개발할 수 있고, 그를 통해 보다 효과적인 비만 예방 및 관리가 가능할 것이다. 수용성과 효과성이 높은 중재 전략을 개발하여 비만이라는 건강 문제에 대한 사회적 인식을 개선하고 이를 예방하고 관리하는 문제 해결의 책임을 개인 뿐 아니라 사회가 함께 부담해야 한다는 점에 대한 합의를 도출해내는 데 본 연구가 이론적 근거가 될 수 있을 것으로 기대한다.

References

1. WHO. Obesity and overweight. Fact sheet N°311. 2012.
2. Ministry of Health & Welfare, Korea Centers for Disease Control & Prevention. Korea Health Statistics 2012: Korea National Health and Nutrition Examination Survey (KNHANES V-3). 2013. (Korean)
3. Korean Statistical Information Service. Children and Adolescent Statistics. 2014. *(Korean, authors' translation)*
4. National Health Insurance Service. Result of National Insurance 12-year Big Data Analysis. National Health Insurance Service Press Release (2014.9). *(Korean, authors' translation)*
5. Chan J. The metabolic syndrome: an Asian perspective. Diabetes Voice 2006;51:18-20.
6. Patel A, Huang KC, Janus ED, Gill T, Neal B, Suriyawongpaisal P, et al. Is a single definition of the metabolic syndrome appropriate? - A comparative study of the USA and Asia. Atherosclerosis 2006;184(1):225-232.

7. Yoon NH, Kwon S. Impact of Obesity on Health Care Utilization and Expenditure. The Korean Journal of Health Economics and Policy 2013;19(2):61-80. (Korean)

8. WHO. Obesity: preventing and managing the global epidemic. WHO Technical Report Series 894. 2000.

9. Health Insurance Policy Institute. Imposition of Charge for Health Risk Factors to Secure Health Coverage Funding. 2012. *(Korean, authors' translation)*

10. Bae NK, Kwon IS, Cho YC. Ten Year Change of Body Mass Index in Korean: 1997~2007. The Korean Journal of Obesity 2009;18(1):24-30. (Korean)

11. Kim M, Kim H. Analyses on the dieters' characteristics and the factors determining diet practice in college. Korean Journal of Human Ecology 2008;17(3):521-532. (Korean)

12. Choi BY, Choi MO. The Survey for Body Shape Recognition Fatness Knowledge and Fatness Stress of College Woman. J Kor Soc Cosm 2008;14(4):1052-1064. (Korean)

13. Kim OS, Yoon HS. Factors Associated with Weight Control Behaviors Among High School Females with Normal Body Weight. JKAN 2000;30(2):391-401. (Korean)

14. Tak YJ, Lee YS, Lee JS, Kang JH. The Trend of Obesity-related Researches in Korea: from 1984 to 2002. The Korean Journal of Obesity 2004;13(1):1-13. (Korean)

15. Hey DW. Development of a Multi-disciplinary Ecological Model for Childhood Obesity [dissertation]. Southern Illinois University; 2004.

16. Sherwood. Epidemiology of Obesity. Healthy Generations 2000;1(2):1-3.

17. Dietz WH, Bland MG, Gortmaker SL, Molloy M, Schmid TL. Policy tools for the childhood obesity epidemic. J Law Med Ethics 2002;30(S3):83-87.

18. Horgen KB, Brownell KD. Confronting the toxic environment: Environmental and public health actions in a world crisis. Handbook of Obesity Treatment. Guilford Press. 2002.

19. French SA, Stroy M, Jeffery RW. Environmental influences on eating and physical activity. Annu Rev Public Health 2001;22:309-335.

20. Berkman L, Kawachi I. Social Epidemiology, 1st ed. Oxford University Press; 2005.

21. Kim YI, Chung HS, Bae KH. Health Promotion Theory and Practice. Seoul: Episteme; 2007. *(Korean, authors' translation)*

22. Martikainen PT, Marmot MG. Socioeconomic differences in weight gain and determinants and consequences of coronary risk factors. Am J Clin Nutr 1999;69(4):719-726.

23. Sundquist J, Malmstrom M, Johansson SE. Cardiovascular risk factors and the neighbourhood environment: a multilevel analysis. Int J Epidemiol 1999;28(5):841-845.

24. Kim MY, Oh JK, Lim MK, Yun EH, Kang YH. The Association of Socioeconomic and Psychosocial Factors with Obesity in a Rural Community. The Korean Journal of Obesity 2012;21(2):18-28. (Korean)

25. Khang YH, Kim HR. Explaining socioeconomic inequality in mortality among South Koreans: an examination of multiple pathways in a nationally representative

longitudinal study. Int J Epidemiol 2005;34(3):630-637.

26. Monteiro CA, Conde WL, Popkin BM. Independent effects of income and education on the risk of obesity in the Brazilian adult population. J Nutr 2001;131:881S-886S.

27. Frank LD, Kerr J, Sallis JF, Miles R, Chapman J. A hierarchy of sociodemographic and environmental correlates of walking and obesity. Prev Med 2008;47:172-178.

28. Feng J, Glass TA, Curriero FC, Stewart WF, Schwartz BS. The built environment and obesity: A systematic review of the epidemiologic evidence. Health & Place 2010;16:175-190.

29. Saelens BE, Glanz K. Work Group I: Measures of the Food and Physical Activity Environment: Instruments. Am J Prev Med 2009;36(4S):S166-S170.

30. Sallis JF, Cervero RB, Ascher W, Henderson KA, Kraft MK. Kerr J. An ecological approach to creating active living communities. Annu Rev Public Health 2006;27:297–322.

31. Kim EJ, Kim HS, Lee SB, Kang MK. Study on the Spatial Planning and Public Policies for Creating a Healthy City. KRIHS. 2010. (Korean)

32. Lee KH, Ahn KH. The Correlation between Neighborhood Characteristics and Walking of Residents – A Case study of 40 Areas in Seoul. Journal of Korean Planners Association 2007;42(6):105-118. (Korean)

33. Brownson RC, Hoehner CM, Day K, Forsyth A, Sallis JF. Measuring the built environment for physical activity: state of the science. American Journal of Prev

Med 2009;36(4):S99–S123.

34. Ding D, Gebel K. Built environment, physical activity, and obesity: What have we learned from reviewing the literature? Health & Place 2012;18:100-105.

35. Gebel K, Bauman AE, Sugiyama T, Owen N. Mismatch between perceived and objectively assessed neighborhood walkability attributes: prospective relationships with walking and weight gain. Health & Place 2011;17(2):519–524.

36. Thow AM, Xuereb G, Randby S. Population-based prevention strategies for childhood obesity [discussion paper presented to the WHO Forum and Technical Meeting on Population-based Prevention Strategies for Childhood Obesity, Geneva, Switzerland, 15–17December2009]. WHO. 2009.

37. Dietz WH, Bland MG, Gortmaker SL, Molloy M, Schmid TL. Policy tools for the childhood obesity epidemic. J Law Med Ethics 2002;30(S3):83-87.

38. Booth S. Environmental and societal factors affect food choice and physical activity: Rationale, Influences, and Leverage Points. Nutr Rev 2001;59(3):21-39.

39. Kim H. Future Directions and Strategies of the Obesity Prevention Policies and Programs. Health and Welfare Policy Forum 2001;173:41-54.

40. Glanz K., Rimer BK., Wiswanath K. Health behavior and health education. Jossey-Bass: 2008.

Tables and Figures

Table 1. Descriptions of variables

Variables	Descriptions	Sources
**Individual factors (Individual level)**		
Socio-demographic characteristics		
Sex	Male, Female	Community health survey
Age	19-34 years, 35-49 years, 50-64 years	
Household income	Household income quartiles	
Education	Middle school or under, High school, College or over	
Occupation	Non-manual, Manual, Others(including students, housewives, not working)	
Marital status	Live with spouse, Live without spouse	
Health behaviors		
Smoking	Current smokers, Former smokers, Never smokers	
Alcohol drinking	More than once a month, Less than once a month	
Physical activity	Moderate physical activity at least 30 minutes 5 times per week or vigorous physical activity at least 20 minutes 3 times per week	
Walking	Regular walking for at least 30 minutes 5 times per week	
Low-sodium diet	Not add salt or soy sauce at the meal	
Sleep duration	Less than 7 hours, 7-8 hours, More than 8 hours	
Stress	Severe, Less severe	
**Environmental factors (Local level)**		
Economical environment		
Population density	1,000 persons per ㎢	Statistics of residence registration population
Fiscal self-reliance ratio	ratio of own-source revenue to total local revenue	Municipal year book of Korea
High education rate	Population rate of university-or- higher	Population

	education level	census
<u>Physical environment</u>		
Park area	Park area per total local area (㎢)	Statistics of urban plan
Exercise facilities	Exercise facilities per 100 persons	Municipal year book of Korea
Pedestrian safety	Illegally parked car share at school zone, pedestrian fatalities per thousand people in traffic accidents	Traffic culture index
Perceived accessibility of exercise facilities	The percentage of participants that perceive the accessibility of exercise facilities as easily	
<u>Social Environment</u>		
Satisfaction with safety	The percentage of participants that satisfy with the general safety (e.g. natural disaster, traffic accident, crime)	
Satisfaction with natural environment	The percentage of participants that satisfy with the natural environment (e.g. air, water)	
Satisfaction with living environment	The percentage of participants that satisfy with the living environment (e.g. electricity, water and sewage, removal of garbage, sports facilities)	Community health survey
Satisfaction with public transportation	The percentage of participants that satisfy with the public transportation system (e.g. bus, subway, taxi)	
Social support	The percentage of participants that trust and help their neighbors	
Nutrition education participation	Nutrition education participation rate at local public health centers, hospitals, or schools	
Exercise program participation	Exercise program participation rate at local public health or service centers	

Table 2. General characteristics of subjects

	BMI<25	BMI≥25	Total	*p*-value
	n (%)	n (%)	n (%)	(χ^2-test)
**Sex**				
Male	108,192 (68.6)	49,637 (31.4)	157,829 (46.8)	<.0001
Female	146,057 (81.5)	33,250 (18.5)	179,307 (53.2)	
**Age**				
19-34 years	68,371 (81.1)	15,913 (18.9)	84,284 (25.0)	<.0001
35-49 years	96,321 (74.7)	32,542 (25.3)	128,863 (38.2)	
50-64 years	89,557 (72.2)	34,432 (27.8)	123,989 (36.8)	
**Household income**				
1st Quartile	30,822 (73.9)	10,865 (26.1)	41,687 (13.2)	<.0001
2nd Quartile	72,662 (74.4)	25,024 (25.6)	97,686 (30.9)	
3rd Quartile	67,732 (75.8)	21,647 (24.2)	89,379 (28.3)	
4th Quartile	66,623 (76.6)	20,335 (23.4)	86,958 (27.5)	
**Education**				
Middle school	56,649 (70.4)	23,765 (29.6)	80,414 (23.9)	<.0001
High school	108,324 (76.8)	32,774 (23.2)	141,098 (41.9)	
College	88,757 (77.2)	26,186 (22.8)	114,943 (34.2)	
**Occupation**				
Non-manual	62,983 (76.2)	19,713 (23.8)	82,696 (24.6)	<.0001
Manual	114,562 (72.6)	43,340 (27.4)	157,902 (46.9)	
Others	76,394 (79.5)	19,731 (20.5)	96,125 (28.5)	
**Marital status**				
Live without spouse	76,737 (80.0)	19,183 (20.0)	95,920 (28.5)	<.0001
Live with spouse	177,283 (73.6)	63,647 (26.4)	240,930 (71.5)	
**Smoking**				
Current smoker	57,654 (70.7)	23,874 (29.3)	81,528 (24.2)	<.0001
Former smoker	30,172 (65.1)	16,148 (34.9)	46,320 (13.7)	
Never smoker	166,371 (79.5)	42,854 (20.5)	209,225 (62.1)	
**Alcohol drinking**				
< once a month	106,684 (77.0)	31,881 (23.0)	138,565 (41.1)	<.0001
≥ once a month	147,489 (74.3)	50,987 (25.7)	198,476 (58.9)	
**Physical activity**				
No	193,778 (75.9)	61,627 (24.1)	255,405 (76.1)	<.0001
Yes	59,441 (73.9)	20,975 (26.1)	80,416 (23.9)	
**Walking**				
No	147,368 (74.8)	49,536 (25.2)	196,904 (58.5)	<.0001
Yes	106,393 (76.2)	33,225 (23.8)	139,618 (41.5)	
**Low-sodium diet**				
No	155,249 (74.9)	51,946 (25.1)	207,195 (61.5)	<.0001
Yes	98,988 (76.2)	30,940 (23.8)	129,928 (38.5)	

**Sleep duration**				
< 7 hours	106,499 (73.4)	38,561 (26.6)	145,060 (43.0)	<.0001
7-8 hours	138,985 (76.9)	41,719 (23.1)	180,704 (53.6)	
≥ 8 hours	8,699 (77.1)	2,581 (22.9)	11,280 (3.3)	
**Stress**				
Less severe	185,367 (76.2)	58,028 (23.8)	243,395 (72.2)	<.0001
severe	68,805 (73.5)	24,839 (26.5)	93,644 (27.8)	
**Total**	254,249 (75.4)	82,887 (24.6)	337,136 (100.0)	

Table 3. Factors affecting the development of obesity

		Model 1			Model 2			Model 3		
		Estimate	SE	Pr > |t|	Estimate	SE	Pr > |t|	Estimate	SE	Pr > |t|
**Fixed effects**										
**Individual level**										
Sex (Ref. Male)	Female				-0.727	0.013	<.0001	-0.714	0.013	<.0001
Age (Ref. 19-34 years)	35-49 years				0.139	0.015	<.0001	0.142	0.015	<.0001
	50-64 years				0.188	0.013	<.0001	0.189	0.013	<.0001
Household Income (Ref. 4th Quartile)	1st Quartile				0.080	0.016	<.0001	0.083	0.017	<.0001
	2nd Quartile				0.069	0.012	<.0001	0.062	0.012	<.0001
	3rd Quartile				0.033	0.012	0.005	0.030	0.012	0.016
Education (Ref. College)	Middle School				0.374	0.015	<.0001	0.366	0.016	<.0001
	High School				0.037	0.011	0.001	0.032	0.012	0.007
Occupation (Ref. Non-manual)	Manual				-0.032	0.014	0.019	-0.032	0.014	0.026
	Others				-0.019	0.012	0.130	-0.021	0.013	0.100
Marital status (Ref. Live with spouse)	Live without spouse				-0.265	0.011	<.0001	-0.262	0.012	<.0001
Smoking (Ref. Never smoker)	Current smoker				-0.040	0.014	0.003	-0.031	0.014	0.031
	Former smoker				0.162	0.015	<.0001	0.173	0.016	<.0001
Alcohol drinking	≥ once a month				-0.046	0.010	<.0001	-0.046	0.010	<.0001
Physical Activity					0.000	0.010	0.965	-0.002	0.011	0.881
Walking					-0.061	0.009	<.0001	-0.062	0.009	<.0001
Low-sodium diet					-0.002	0.009	0.793	-0.003	0.010	0.741
Sleep duration (Ref. 7-8 hours)	< 7 hours				0.155	0.009	<.0001	0.159	0.009	<.0001
	≥ 8 hours				0.033	0.025	0.180	0.034	0.026	0.191
Stress (Ref. Less severe)	Severe				0.143	0.009	<.0001	0.153	0.010	<.0001
**Local level**										

Population density							0.004	0.002	0.052
Fiscal self-reliance ratio							0.068	0.076	0.371
High education rate							-0.405	0.152	0.008
Park area							-0.854	0.490	0.082
Exercise facilities							0.083	0.097	0.395
Pedestrian safety							-0.018	0.060	0.760
Perceived accessibility of exercise facilities							-0.152	0.090	0.091
Satisfaction with safety							-0.370	0.216	0.088
Satisfaction with natural environment							0.266	0.127	0.037
Satisfaction with living environment							0.057	0.160	0.723
Satisfaction with public transportation							-0.298	0.087	0.001
Social support							-0.029	0.062	0.643
Nutrition education participation							0.511	0.285	0.073
Exercise program participation							0.172	0.242	0.477
**Random effects**									
σ^2	0.022	0.002	<.0001	0.013	0.002	<.0001	0.009	0.001	<.0001

Table 4. Factors affecting the development of obesity according to the sex

		Male						Female					
		Model 2			Model 3			Model 2			Model 3		
		Estimate	SE	Pr > |t|	Estimate	SE	Pr > |t|	Estimate	SE	Pr > |t|	Estimate	SE	Pr > |t|
**Fixed effects**													
**Individual level**													
Age (Ref. 19-34 years)	35-49 years	-0.198	0.020	<.0001	-0.198	0.021	<.0001	0.464	0.024	<.0001	0.475	0.025	<.0001
	50-64 years	-0.010	0.017	0.574	-0.009	0.018	0.605	0.367	0.021	<.0001	0.368	0.022	<.0001
Household Income	1st Quartile	-0.086	0.022	0.000	-0.080	0.024	0.001	0.247	0.023	<.0001	0.230	0.024	<.0001
(Ref. 4th Quartile)	2nd Quartile	-0.039	0.016	0.013	-0.045	0.016	0.007	0.219	0.019	<.0001	0.205	0.020	<.0001
	3rd Quartile	-0.034	0.015	0.025	-0.036	0.016	0.022	0.125	0.019	<.0001	0.118	0.020	<.0001
Education (Ref. College)	Middle School	-0.069	0.020	0.001	-0.085	0.021	<.0001	0.814	0.024	<.0001	0.804	0.026	<.0001
	High School	-0.083	0.014	<.0001	-0.093	0.015	<.0001	0.332	0.019	<.0001	0.328	0.020	<.0001
Occupation	Manual	-0.269	0.023	<.0001	-0.262	0.024	<.0001	0.192	0.022	<.0001	0.193	0.023	<.0001
(Ref. Non-manual)	Others	-0.063	0.015	<.0001	-0.064	0.016	<.0001	0.128	0.023	<.0001	0.123	0.024	<.0001
Marital status	Live without spouse	-0.283	0.016	<.0001	-0.283	0.017	<.0001	-0.262	0.017	<.0001	-0.251	0.018	<.0001
Smoking	Current smoker	-0.030	0.015	0.046	-0.015	0.016	0.337	0.051	0.037	0.171	0.051	0.039	0.186
(Ref. Never smoker)	Former smoker	0.240	0.016	<.0001	0.255	0.017	<.0001	0.263	0.045	<.0001	0.263	0.047	<.0001
Alcohol drinking	≥ once a month	0.030	0.014	0.034	0.025	0.015	0.091	-0.092	0.014	<.0001	-0.087	0.014	<.0001
Physical Activity		-0.002	0.013	0.877	0.000	0.014	0.972	-0.027	0.016	0.102	-0.038	0.017	0.026
Walking		-0.084	0.012	<.0001	-0.081	0.012	<.0001	-0.062	0.014	<.0001	-0.068	0.014	<.0001
Low-sodium diet		-0.036	0.013	0.005	-0.037	0.013	0.007	0.037	0.013	0.005	0.036	0.014	0.011
Sleep duration	< 7 hours	0.142	0.012	<.0001	0.148	0.012	<.0001	0.102	0.013	<.0001	0.104	0.014	<.0001
(Ref. 7-8 hours)	≥ 8 hours	0.032	0.036	0.375	0.043	0.038	0.256	0.152	0.034	<.0001	0.145	0.036	<.0001
Stress (Ref. Less severe)	Severe	0.077	0.013	<.0001	0.087	0.013	<.0001	0.193	0.014	<.0001	0.203	0.015	<.0001
**Local level**													

Population density				0.004	0.002	0.069				0.004	0.003	0.103
Fiscal self-reliance ratio				-0.046	0.089	0.606				0.195	0.103	0.059
High education rate				0.025	0.176	0.888				-1.072	0.209	<.0001
Park area				-0.375	0.565	0.507				-1.501	0.668	0.025
Exercise facilities				0.081	0.113	0.470				0.091	0.131	0.487
Pedestrian safety				-0.031	0.070	0.662				-0.011	0.080	0.892
Perceived accessibility of exercise facilities				-0.067	0.112	0.549				-0.270	0.125	0.032
Satisfaction with safety				-0.594	0.251	0.019				-0.089	0.288	0.758
Satisfaction with natural environment				0.294	0.148	0.047				0.275	0.170	0.107
Satisfaction with living environment				0.110	0.188	0.561				-0.075	0.215	0.727
Satisfaction with public transportation				-0.242	0.101	0.017				-0.332	0.116	0.005
Social support				0.082	0.072	0.255				-0.144	0.083	0.084
Nutrition education participation				0.743	0.364	0.041				0.399	0.409	0.329
Exercise program participation				0.036	0.305	0.906				0.399	0.345	0.248
**Random effects**												
σ^2	0.013	0.002	<.0001	0.010	0.002	<.0001	0.023	0.003	<.0001	0.013	0.002	<.0001
